# Effectiveness of the High Dose/Refuge Strategy for Managing Pest Resistance to *Bacillus thuringiensis *(*Bt*) Plants Expressing One or Two Toxins

**DOI:** 10.3390/toxins4100810

**Published:** 2012-10-18

**Authors:** Aiko Gryspeirt, Jean-Claude Grégoire

**Affiliations:** 1 Biological Control and Spatial Ecology Laboratory (LUBIES), CP 160/12, Université Libre de Bruxelles, av. FD Roosevelt 50, B-1050 Brussels, Belgium; Email: jcgregoi@ulb.ac.be; 2 Research Funding for Industry and Agriculture (FRIA, Fonds pour la Formation à la Recherche dans l’Industrie et l’Agriculture), 5 rue d’Egmont, B-1000 Brussels, Belgium

**Keywords:** insect resistance management, population dynamics, population genetics, *Bacillus thuringiensis*, High dose/Refuge strategy, *Bt *plants, two toxins

## Abstract

To delay resistance development to *Bacillus thuringiensis* (*Bt*) plants expressing their own insecticide, the application of the Insect Resistance Management strategy called “High Dose/Refuge Strategy” (HD/R) is recommended by the US Environmental Protection Agency (US EPA). This strategy was developed for *Bt *plants expressing one toxin. Presently, however, new *Bt* plants that simultaneously express two toxins are on the market. We used a mathematical model to evaluate the efficiency of the HD/R strategy for both these *Bt* toxins. As the current two-toxin *Bt* plants do not express two new Cry toxins but reuse one toxin already in use with a one*-*toxin plant, we estimated the spread of resistance when the resistance alleles are not rare. This study assesses: (i) whether the two toxins have to be present in high concentration, and (ii) the impact of the relative size of the refuge zone on the evolution of resistance and population density. We concluded that for *Bt* plants expressing one toxin, a high concentration is an essential condition for resistance management. For the pyramided *Bt* plants, one toxin could be expressed at a low titer if the two toxins are used for the first time, and a small refuge zone is acceptable.

## 1. Introduction

*Bacillus thuringiensis* (*Bt*) was discovered in 1901 in Japan in diseased silkworms and has been produced on an industrial scale for its insecticidal properties since 1938; however, only in 1953 was it shown that its insecticidal properties were the result of the parasporal inclusion of a crystal protein called Cry toxin [1]. Unlike many synthetic insecticides which traverse the integument and act on the insect’s nervous system, these toxins have to be ingested [2]. The proteolytically activated Cry toxin binds to specific receptors on the brush border membrane of the midgut. Pores are formed and the affected cells swell and are lysed. The death of the insect follows within hours or days [3]. These insecticidal proteins are active ingredients of some environmentally friendly insecticides because they are harmless to natural enemies and other nontarget organisms due to their narrow host specificity and short life in the environment [4]. Today, *Bacillus thuringiensis* is considered the most successful insect pathogen and presently constitutes about 2% of the total insecticidal market [5].

A Cry gene was first introduced into tobacco and tomato plants in 1987. These transgenic hybrids expressing the insecticidal toxin (*Bt *plants) are able to withstand insect attack due to exceptional expression levels of a Cry toxin [6,7]. Introduced on the market in 1996, *Bt *plants expressing one toxin covered about 50 million hectares (ha) worldwide in 2010 and have been planted on >200 million hectares (ha) since 1996. More precisely, *Bt *corn and *Bt *cotton covered 42 million ha in 2007 and their target insect are Lepidoptera and Coleoptera [8]. One risk linked to this extensive use is an increasing selection of resistant insects to *Bt* plants, which could limit the use of *Bt* plants in time and space [9]. 

To delay and manage resistance outbreaks in the field environment, a frequent solution is to develop and apply Insect Resistance Management (IRM) strategies. These preventive strategies have to be applied before resistance spreads widely in the population. Numerous IRM strategies have been proposed: (1) mixtures, mosaics or rotations of transgenic plants; (2) time- or tissue-specific toxin expression; (3) low doses of toxins in combination with natural enemies; (4) co-expression of different Cry genes; (5) high expression of the toxin (dose) with refuge of non-*Bt* plants [9]. This last strategy, the so-called “High Dose/Refuge Strategy” (HD/R), requires planting a “refuge zone” composed of non-*Bt* plants suitable for the target pest and in close proximity to a “*Bt* zone” producing a high concentration of Cry toxin (25 to 50 times the LD99) [10].

The HD/R strategy assumes that resistance is an autosomal trait controlled by a single locus with two alleles (the rare resistance allele: Freq Ar < 10^−3^—the susceptible allele: As), with no sex linkage and no maternal effect, as largely supported experimentally [11,12,13]. Susceptible adults emerge from the refuge zone and resistant homozygotes (ArAr) from the *Bt* zone. The assumptions of this strategy are that mating is panmictic and egglaying is random between the refuge and the *Bt* zones. If inheritance of resistance is functionally recessive, heterozygous progeny produced by such matings will be killed by ingestion of high concentration toxin in the *Bt* zone at the same rate as homozygous susceptible larvae. By killing heterozygotes the high dose crop can greatly slow down the increase of the resistance allele frequency.

A substantial number of studies have evaluated the impact of these strategies on resistance development. A key tool in this field is mathematical or computer modelling, and various models have played an essential role in building a conceptual framework for resistance management [14,15,16,17,18,19].

Today, *Bt *cotton and *Bt *maize still dominate the market and the new hybrids registered in the United States collectively produce 18 different combinations of 11 *Bt *toxins [20]. The second-generation *Bt *plants simultaneously produce two dissimilar insecticidal toxins active against the same pest [21]. This “pyramidal” approach has been on the market since 2002. US EPA has registered Bollgard II^®^cotton (Cry1Ac in combination with Cry2Ab2; Monsanto) in 2002, Widestrike^®^cotton (Cry1F and Cry1Ac; Dow Agro-sciences) in 2004 and, MON 810 × TC1507 Corn (Cry1Ab and Cry1F; Pioneer/Dupont) in 2010 [21]. The dual-toxins plants provide a substantially better control of insect pests [22]. They should delay the spread of resistance when selection for resistance to one of the toxins does not cause crossresistance to the other toxin because resistance to each toxin is initially rare and cases of double resistance will be extremely infrequent. This was demonstrated by theoretical models and by experiments [23,24,25,26,27,28,29,30,31]. However, IRM strategies remain essential because dual resistance should be triggered in large populations by uniform and long-term simultaneous exposure to the two toxins over large areas [32]. The widespread adoption of these transgenic varieties has thus greatly increased the opportunity for resistance selection in the field [33].

Initially, the HD/R strategy was developed for one-toxin *Bt* plants. This study evaluates its efficiency for pyramided *Bt* plants. 

In Australia, Bollgard^®^ was directly replaced in 2004/2005 with Bollgard II^®^. In the USA, during the transition to the second-generation Bollgard II^®^, both the single- and two-toxin *Bt* cottons have been grown since 2003. The process ended in 2010, since the US EPA registration for Bollgard I^®^ cotton expired at the end of 2009. However in India, Bollgard^®^ and Bollgard II^®^ coexists since 2006 [34,35]. Zhao and colleagues [30] studied the impact of the concurrent use of *Bt* plants expressing either one single, or two Cry toxins, and sharing one of these. They concluded that this procedure selects for resistance to two-toxin *Bt* plants more rapidly than the use of two one-toxin *Bt *plants alone. Therefore, two toxin *Bt* plants should not be deployed simultaneously with one-toxin *Bt* plants if they share one similar Cry toxin. With our model, we evaluate the spread of resistance when a two-toxin *Bt *crop is planted alone after a one-toxin *Bt *crop (sharing a similar Cry toxin). In this case, the resistance allele frequency to the shared toxin should not be rare (>10^−3^) if the resistance is developed for the toxin already used [12].

One essential condition of the HD/R strategy is the high toxin concentration in the *Bt *plant. In this study, we assess if this condition is necessary for the pyramided *Bt *plants.We also measure the impact of the relative size of the refuge zone on changes in resistance and in the population density in our study area. 

For these purposes, a deterministic and discrete time model in two parts was developed. The first part is based on population genetics theory and allows us to follow the frequency of resistance at each generation. The second is a population dynamics model, which simulates population changes. This last part is essential to assess the impact of an IRM strategy on pest population density because controlling the pest populations does, in itself, limit crop damages.

## 2. Materials and Methods

### 2.1. Parameters

The effectiveness of any IRM strategy is related to two kinds of parameters: the operational parameters characterizing the strategy and the biological parameters characterizing the target insect [36]. The operational parameters include the relative size of the refuge zone (**v**) in relation to the whole field and the selection pressure exerted by the Cry toxin on susceptible insects (**s*Bt*A**, **s*Bt*B**). The biological parameters are the initial frequency of the resistance allele for each locus (**ArFreq**, **BrFreq**) and its dominance (**hAr**, **hBr**), the fitness cost associated with resistance (**fcostA**, **fcostB**) and its dominance (**hfcA**, **hfcB**), the initial population size (**nzero**), the population intrinsic growth rate (**r**) and the field carrying capacity (**K**). Each parameter is associated with a default value taken from the literature and measured on a lepidopteran insect pest (see Table 1).

**Table 1:** Operational and biological parameters introduced in the simulation model: their symbol, default and tested values.

		Symbol	Default-Value	Tested-Value
**Operational Parameters**	Refuge zone proportion	**v**	0.05 [37]	0.05–0.10–0.20–0.30–0.40
	Selection of the toxin A	*sBtA*	1 [37]	1–0.93–0.50
	Selection of the toxin B	*sBtB*	1 [37]	1–0.93–0.50
	Field area (hectare)		260 [38]	na
	Plants/hectare		67,000 [38]	na
**Biological Parameters**	Initial Ar frequency	*ArFreq*	1.5 × 10^−3^ [39]	1.5 × 10^−2^–1.5 × 10^−1^
	Initial Br frequency	BrFreq	1.5 × 10^−3 ^ [39]	1.5 × 10^−2^–1.5 × 10^−1^
	Ar dominance	*hAr*	0 [12,40]	0–0.23–0.53
	Br dominance	*hBr*	0 [12,40]	0–0.23–0.53
	Fitness cost associated to Ar	*fcostA*	0.15 [41]	na
	Fitness cost associated to Br	*fcostB*	0.15 [41]	na
	Fitness cost dominance associated to Ar	*hfcA*	0 [42,43,44]	na
	Fitness cost dominance associated to Br	*hfcB*	0 [42,43,44]	na
	Initial individual number/ha	*nzero*	50,000 [38]	na
	Intrinsic growth rate	*r*	0.15 [45,46]	na
	Carrying capacity/plant	*K*	22 [38]	na

#### 2.1.1. Resistance Alleles

There is one single locus associated with resistance for each of the two toxins (toxin A and toxin B). Each locus is characterized by two alleles: the resistant and the susceptible allele (Ar–As and Br–Bs) and we assumed that there was no linkage.

Among the large number of insecticidal Cry many exhibit cross-resistance (so, resistance to one toxin confers an increased resistance to a second Cry toxin [47]). *Heliothis virescens *(Lepidoptera: Noctuidae) (Fabricius) individuals selected with one single type of Cry toxin also displayed a broad-spectrum *Bt *resistance. There was a non-surprising crossresistance between Cry1Ac and Cry1Ab (these toxins are similar in structure and toxicity) and an unexpected crossresistance between Cry1Ac and Cry2A (the amino acid sequences are very different and the toxins differ in their mode of action) [48]. Crossresistance between Cry1Ab and Cry1Ac is also recorded for *Plodia interpunctella *(Lepidoptera: Pyralidae) (Hübner) [49]. Generally, complete crossresistance (selection with one toxin leads to an identical change in resistance levels of a second toxin) is possible but in many instances, selection with a toxin only confers partial crossresistance to other toxins [48]. The combination of the toxins has to be approached very carefully for an appropriate resistance management [28]. In this study, we do not consider the crossresistance

As an initial condition, the resistance allele frequency is low (ArFreq = BrFreq: 0.0015) but the efficiency of the HD/R strategy is evaluated when resistance is not rare (ArFreq, BrFreq: 0.015 or 0.15).

#### 2.1.2. Relative Size of the Refuge Zone (v)

Theoretically, a refuge zone should produce 500 susceptible insects for every resistant insect that survives on a *Bt* crop [50]. Determining the optimally efficient size of the refuge zones, their arrangement and their acceptability for the users or sellers of *Bt* crops is, today, a matter of some debate [51]. Cry1Ac cotton growers of the USA have to choose among different structural options: 5% external and unsprayed refuge; 20% external and sprayed refuge and 5% embedded refuge [52]. On the other hand, Monsanto Company has petitioned the US EPA to eliminate the refuge where BollgardII^®^ cotton is planted, as natural refuge is assumed enough to maintain susceptible insects [53]. The US EPA has approved this alternative [54]. In our study, we only consider the externally-structured and unsprayed refuge *v* with a default value of 5% in our standard conditions but other values (10%, 20%, 30% and 40%) are tested.

#### 2.1.3. Mortality Exerted by the *Bt* Plants on Susceptible Insects and Resistance Allele Dominance

According to the initial assumptions of HD/R strategy, *Bt* plants produce high toxin titer (*sBtA* = 1) and the resistance allele is recessive (*hAr* = 0). This will eliminate the heterozygous offspring and slow down the increase of the Ar frequency [9], because when an allele is rare, it occurs almost exclusively in the heterozygous state. Therefore, during early generations, the resistance allele dominance is the main determinant of the rate of evolution [36]. Basically, the HD/R strategy requires a resistance controlled by a recessive single locus with two alleles (As-Ar) [11,12,13]. In most cases studied so far, the inheritance of high levels of resistance to *Bt* toxins in field- and laboratory- selected populations is recessive [55,56]. Although the general consensus is that only the operational factors can be manipulated [36], experimental data show relationships between *Bt* toxin concentration, larval mortality and functional dominance [11,40,57,58]. Dominance of resistance is not a fixed value and presents a plastic response depending on environmental parameters: the recessivity of resistance is associated with more demanding environments [59]. Resistance to Cry1Ac in *Pectinophora gossypiella* (Lepidoptera: Gelechiidae) (Saunders) is codominant at low concentrations (*sBtA *= 0.5; *hAr *= 0.53), partially recessive at middle concentrations (*sBtA* = 0.93; *hAr* = 0.23) and completely recessive at high concentrations (*sBtA *= 1, *hAr* = 0) [13]. These three situations are evaluated in this study to assess the impact of the toxin concentration when *Bt *plants express two toxins simultaneously: 1/*sBtA *or *sBtB*: *1*; *hAr *or *hBr*: 0 (recessive), 2/*sBtA *or *sBtB*: 0.93; *hAr *or *hBr*: 0.23 (partially recessive) and 3/*sBtA *or *sBtB*: 0.50; *hAr *or *hBr*: 0.53 (codominant).

#### 2.1.4. Fitness Cost of Resistance and Fitness Cost Dominance

Selection for any novel trait can induce changes correlated with other characters, mainly through the ability of a single gene to affect more than one trait (pleiotropy) [60]. This antagonistic trade-off in fitness caused by the presence of a resistance gene will be referred as a “fitness cost.” Here, we suppose that it only affects survival. The fitness cost and its dominance are central for predicting the behavior of the resistance gene because the cost increases the advantage of SS over RS individuals in refuges and favors the decline of resistance [61]. The default values of these two parameters are the same as in simulations of Tabashnik *et al*. [42] and of Vacher *et al*. [62]: *fcostA *= *fcostB *= 0.15 and *hfcA = hfcB *= 0. 

#### 2.1.5. Fitness Estimation

The fitness is the × ability to survive and reproduce, and thus have offspring in future generations [47]. Its value is associated with the genotype (ArAr, ArAs, AsAs for toxin A or BrBr, BrBs, BsBs for toxin B) and to the environment. Fitness is calculated for each genotype in the *Bt* or refuge zone following the Lenormand equations (equation 1) [63].


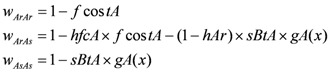
(1)

The function gA(*x*) equals one if the insect is on *Bt* plants expressing the A toxin and equals zero otherwise. The parameter *hAr *is the dominance level of the resistance allele associated with toxin selection [62]. Similar considerations are made for the B toxin.

As there is no crossresistance between the two toxins and no pleiotropic effect, the fitness of the genotype of the locus A is independent of the fitness of the genotype of the locus B. Thus, with two-toxin *Bt* plants, the global fitness is the product of the fitness of both genotypes (e.g.: w ArAr_BrBr = w ArAr × w BrBr).

#### 2.1.6. Initial Individual Number (Nzero); Carrying Capacity (K); Population Intrinsic Growth Rate (r):

For the values of the parameters characterizing the population dynamics in this system, we use the published data concerning the main Lepidopteran pests (see Table 1). *K* and *r *are independent of the zone in the field (*Bt *or refuge zone), and of the genotype [45,46].

### 2.2. Model Description

**Figure 1:**
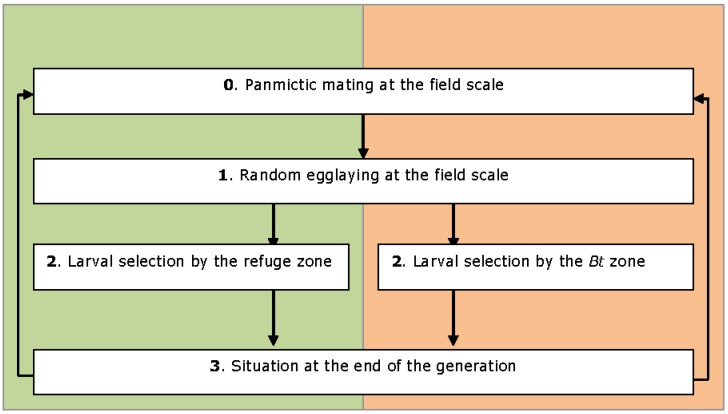
Stages composing the model.

The considered system is a section of a homogeneous region of 260 ha, as proposed by Guse *et al. *[38]. This field is closed and composed of two adjacent zones: the *Bt* zone and the refuge zone. The insect population is uniformly distributed among the zones and cannot leave the area. Each generation is divided into a succession of simple stages and the generations are discrete and continuous (*i.e.*, no diapause) (see Figure 1). The time unit is the non-overlapping generation.

At step 0, there is panmictic mating at the field scale followed by random egglaying between the two habitats (Step 1). Oviposition choice tests indicate that females are unable to discriminate between the two plants varieties and show no oviposition preferences [64,65]. There is no hatching difference between the two zones [64]. After eclosion, the larvae start feeding and are subjected to selection pressure according to their genotypes and to the zone in which they develop (Step 2). Toxin exposure occurs only for larvae. Next, the model calculates the genotype frequencies and the population sizes (Step 3). The adults emerge and the cycle starts again at Step 0. Subject to the condition of panmictic mating and random egglaying, the genotype frequency is present in Hardy-Weinberg proportions in each zone at the beginning of each generation [47]. This Hardy–Weinberg equilibrium is disturbed from the first step of selection but will be restored at Step 0 in the next generation. There is an equation for each stage, implemented in R [66].

Usually, one prerequisite of the refuge theory is that the resistance level of the pool of susceptible insects from a refuge must remain unaffected by an outward migration from the *Bt* zone population [67]. Our model is more realistic: the random egglaying of the resistant females allows the introduction of resistant offspring in the refuge; thus, the refuge is not 100% susceptible but can be “polluted” by resistant alleles.

The first part of this model is based on population genetics theory and allows us to follow the Ar frequency at each generation for one-toxin *Bt *plant or the resistance haplotype frequency (ArBr) for a two-toxin *Bt *plant. This model is inspired by the model of Mallet and Porter [68] and adapted from the equation of Crow and Kimura [69], which indicates the direction of change in the resistance allele frequency at each generation (*t*).

The second part of the model is based on population dynamics theory and allows us to follow the quantitative population changes. Here, reproduction is density-dependant and the population growth follows Ricker’s formulation of a classic discrete logistic population model (equation 2) [70]. The number of pests at time *N*_(*t*+1)_ depends on the population size at time *N*_(*t*)_, on the intrinsic rate of growth (*r*) and on the carrying capacity (*K*).


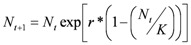
(2)

### 2.3. Model Output

The model investigates the effect of each parameter on the evolution of both resistance and population size. Two indicators are used as standards for comparing simulations.

The most common indicator of resistance spread is the number of generations required to reach 50% frequency of the resistance allele Ar for *Bt* plants expressing one toxin and 50% frequency of the resistant haplotype ArBr for the pyramided plants (GF50) [36,68]. This is a convenient measure of resistance, which is independent of assumptions regarding population growth [31].

The second indicator is the percentage of population decrease between the first two generations and after ten generations. Such an indicator is faster to obtain: only ten generations are required. Secondly, it can be positive or negative, indicating that the population density is increasing or decreasing, respectively, making it more easily interpretable. We consider that the impact of the IRM strategy on population density must be assessed, because it is an important indicator of the sustainability of the strategy.

## 3. Results and Discussion

### 3.1. Efficiency of the HD/R Strategy for *Bt* Plants Expressing High Dose of One or Two Toxins, with Initially Rare Resistance Alleles

**Figure 2:**
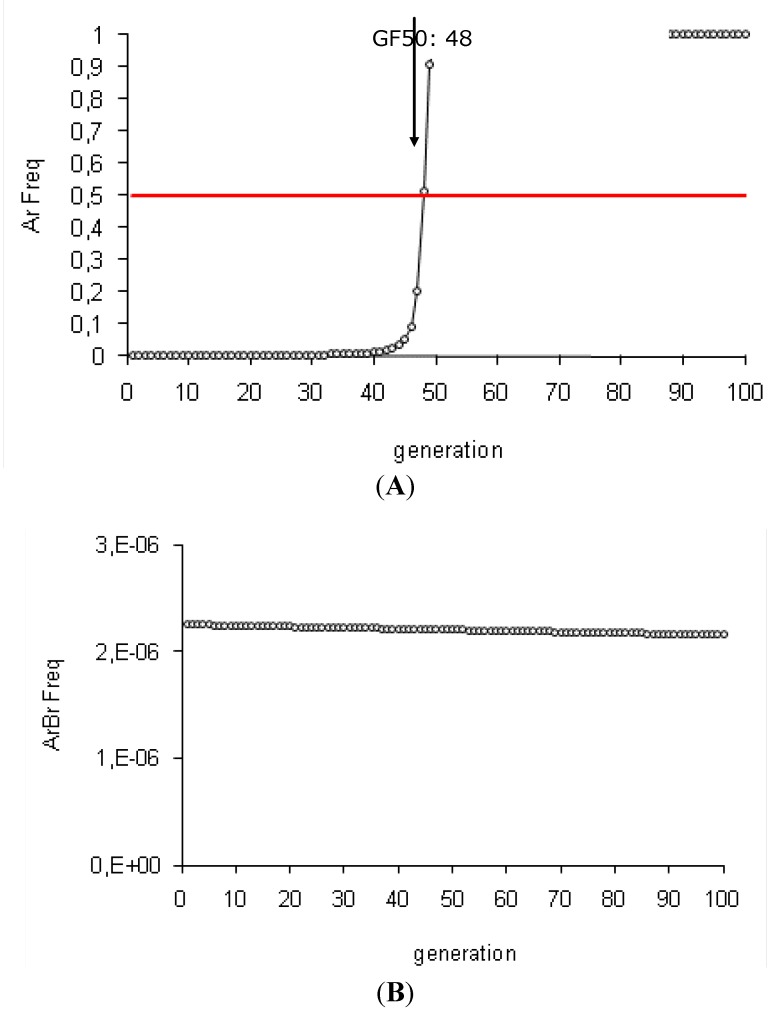
Efficiency of the HD/R strategy with *Bt* plants expressing one or two toxins (initial conditions). These simulations are performed for *Bt *plants expressing one or two toxins with the initial conditions: high toxin concentration (*sBtA* = *sBtB* = 1), recessive resistance allele (*hAr* = *hBr* = 0), initially rare resistance alleles (Ar Freq = Br Freq = 0.0015) and a 5% refuge zone.

Our simulation results are consistent with the HD/R strategy assumptions, with published theoretical models and experimental data [9,23,24,25,26,27,31,71]. The spread of resistance in the population is efficiently delayed by *Bt *plants producing one toxin with a high concentration level. The critical threshold of 50% resistance allele frequency in the population (GF50) is reached at the 48th generation (see Figure 2A and Table 2A). According to our results, this strategy is more effective with *Bt *plants expressing simultaneously two toxins because resistance is reversed in the population (see Table 2B). Only double homozygotes (ArArBrBr) are resistant and resistance to two toxins is a rare event (initially ArArBrBr frequency: 5.06 × 10^−12^ and ArAr frequency: 2.25 × 10^−6^). The resistance alleles are mainly present in the heterozygote insects which are eliminated by the toxins. This induces a decrease of the resistance allele frequency in time. The control of the pest population is similar for *Bt* plants with one or two toxins: there is a reduction of 94% of the population between the first two generations and a elimination of the population after ten generations (100% population decrease between the tenth and the first generation (see Table 2A,B).

### 3.2. Efficiency of the HD/R Strategy if Resistance Is Initially Not Rare in the Population

There is no guarantee that the frequency of the resistance allele is rare. In Arizona, a field population of *Pectinophora gossypiella *(Lepidoptera: Gelechiidae) (Saunders) presented in 1997 an estimated mean frequency of a major resistance allele to Cry1Ac *Bt* cotton ca. 100 times higher than the theoretical threshold [12]. In the southeastern United States, 14 field populations of *Helicoverpa zea* (Lepidoptera: Noctuidae) (Boddie) had more than a hundred-fold higher than theoretical resistance to Cry1Ac *Bt* cotton, and showed a survival increase on leaves of *Bt* cotton plants [20,72,73,74]. Similarly, high resistance reports have been made for *Spodoptera frugiperda *(Lepidoptera: Noctuidae) (Smith) to Cry1F *Bt* corn in Puerto Rico and *Busseola fusca *(Lepidoptera: Noctuidae) (Fuller) to Cry1Ab *Bt *corn in South Africa [75,76].

Resistance to one-toxin *Bt* plants can be problematic for the pyramided *Bt *plant because two-toxin *Bt *plants can have the same Cry toxin as the previous crop of one-toxin *Bt *plants (BollgardII^®^cotton produced Cry1Ac and Cry2Ab derives from BollgardI^® ^cotton protected by Cry1Ac). We consider this last situation in our model, with the complementary hypothesis that, due to previous exposure to one of the toxins, the resistance allele to this toxin is not rare. 

**Figure 3:**
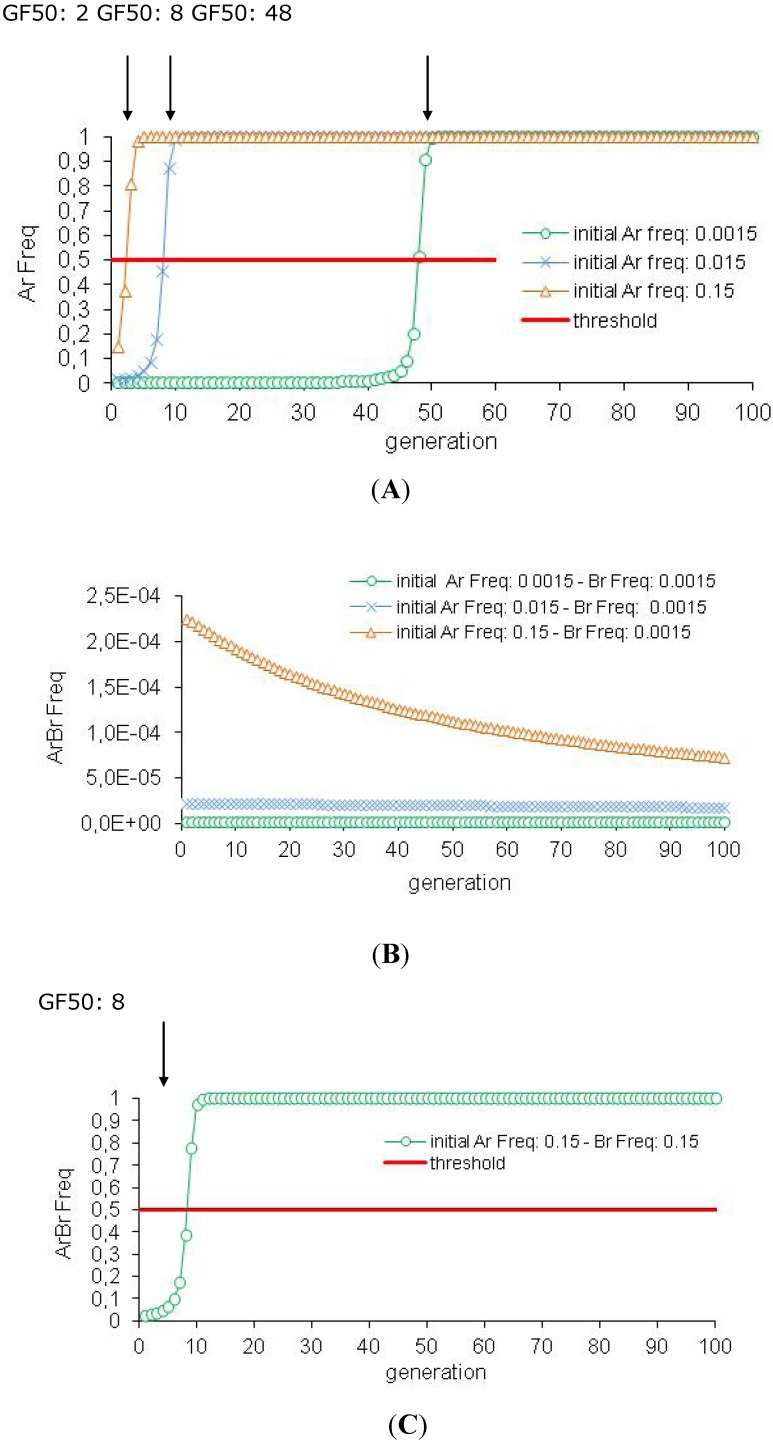
Efficiency of the HD/R strategy with *Bt* plants expressing one or two toxins when the resistance is not rare in the pest population. These simulations are performed for *Bt *plants expressing one or two toxins with a high toxin concentration (*sBtA* = *sBtB* = 1), a recessive resistance allele (*hAr* = *hBr* = 0) and a 5% refuge zone. However, the resistance alleles are not initially rare (Ar Freq and Br Freq = 0.0015, 0.015 or 0.15).

For the one-toxin *Bt *plants, the initial frequency of the resistance allele has a high impact on resistance spread in the population (see Table 2A, Figure 3A). When the resistance allele is not rare, the efficiency of the resistance control drastically decreases. 48 generations are necessary to reach GF50 with an initially rare resistance allele (Ar Freq: 0.0015). This threshold is reached after eight generations for a 0.015 Ar frequency and after two generations for a 0.15 Ar frequency. Our results validate that the HD/R strategy is a preventive strategy, which is only suitable with initially rare resistance alleles [31,32]. The control of pest density is also influenced by the initial allele frequency (see Table 2A). After two generations, there is a 94% population decrease with 0.0015 and 0.015 initial Ar frequencies but a 92% population decrease with a 0.15 initial Ar frequency. However, whatever the initial resistance allele frequency, the insect population is eradicated after ten generations.

**Table 2:** Evolution of resistance and population density.

**A.**	***Bt* plants Synthesizing One Toxin**			**GF50**	**% Pop. Decrease**	
					**Gen 2/Gen1**	**Gen 10/Gen 1**
**1**	**Initial Conditions **(*sBtA* = 1, *hAr* = 0, Ar Freq = 0.001, v = 5%)			48	94	1
**2**	**Impact of the Ar Frequency**	Ar Freq 0.0015		48	94	1
		Ar Freq 0.015		8	94	99.9
		Ar Freq 0.15		2	92	99.1
**3**	**Impact of the Cry Concentration**	hAr 0-sBtA 1		48	94	1
		hAr 0.23-sBtA 0.93		7	86	99.9
		hAr 0.53-sBtA 0.50		20	39	98.7
**4**	**Impact of the Refuge Zone Proportion**	v 0.05		48	94	1
		v 0.1		96	88	99.9
		v 0.2		213	77	99.9
		v 0.3		373	65	98.9
		v 0.4		<500	54	99.8
**B.**	**Bt plants synthesizing two toxins**			**GF50**	**% pop. decrease**	
					**Gen 2/Gen1**	**Gen 10/Gen 1**
**1**	**Initial Conditions**			>500	94	1
**2**	**Impact of the Ar Frequency** **(Br: 0.0015)**	Ar Freq 0.0015		>500	94	1
		Ar Freq 0.015		>500	94	1
		Ar Freq 0.15		>500	94	1
**3**	**Impact of the Ar and Br Frequencies**	Ar Freq 0.15-Br Freq 0.15		8	94	99.9
**4**	**Impact of the Cry** **concentration**	hAr 0-sBtA 1	hBr 0-sBtB 1	>500	94	1
			hBr 0.23-sBtB 0.93	>500	94	1
			hBr 0.53-sBtB 0.50	107	94	1
		hAr 0.53-sBtA 0.50	hBr 0.23-sBtB 0.93	22	90	99.9
			hBr 0.53-sBtB 0.50	25	67	99.9
		hAr 0.23-sBtA 0.93	hBr 0.23-sBtB 0.93	21	94	1
**5**	**Impact of the Cry Concentration and of the Resistance Alleles Frequency**	hAr 0-sBtA 1 hBr 0.23-sBtB 0.93	Ar Freq 0.0015-Br Freq 0.15	>500	94	1
			Ar Freq0.15-Br Freq 0.0015	9	94	q
		hAr 0-sBtA 1 hBr 0.53-sBtB 0.50	Ar Freq 0.0015-Br Freq 0.15	90	94	1
			Ar Freq0.15-Br Freq 0.0015	20	93	99.9
**6**	**Impact of the Toxin Concentrations and of the Refuge Zone Proportion**	hAr 0-sBtA 1 hBr 0.53-sBtB 0.50	v 0.05	107	94	1
			v 0.1	207	88	99.9
			v 0.2	462	77	99.9
			v 0.3	>500	65	99.9
			v 0.4	>500	54	99.8
		hAr 0.53-sBtA 0.50 hBr 0.53-sBtB 0.50	v 0.05	25	67	99.9
			v 0.1	29	62	99.9
			v 0.2	38	53	99.9
			v 0.3	51	45	99.5
			v 0.4	72	36	98.2
		hAr 0.53-sBtA 0.50 hBr 0.23-sBtB 0.93	v 0.05	22	90	99.9
			v 0.1	27	85	99.9
			v 0.2	39	73	99.9
			v 0.3	54	62	99.9
			v 0.4	75	51	99.8
		hAr 0.23-sBtA 0.93 hBr 0.23-sBtB 0.93	v 0.05	21	94	1
			v 0.1	38	87	99.9
			v 0.2	81	76	99.9
			v 0.3	141	65	99.9
			v 0.4	233	53	99.8

For the *Bt *plants expressing two toxins, resistance management remains effective when only one resistance allele is not rare in the population (see Figure 3B and Table 2B). The high toxin concentration (s*Bt*A = s*Bt*B: 1) eliminates all heterozygotes, whereas the homozygous resistant insects (ArArBrBr) are very rare even if one resistance allele is not infrequent (0.0015^2^ * 0.15^2^). In these cases, resistance frequency decreases in the population (so, the GF50 threshold is never reached) and there is a 94% population decrease after two generations and an elimination of the pest population after ten generations. When the two resistance alleles are not rare (Ar = Br frequency: 0.15), resistance spread is rapid (GF50: 8) but the population density is still well controlled (94% and 99.9% population decrease after two and ten generations, respectively) (see Table 2B and Figure 3C). Therefore, although the initial resistance allele frequency for one- or two-toxin *Bt* plants does not seem to influence population density, it can nonetheless have a catastrophic impact on resistance control if the two toxins are not initially rare.

### 3.3. Efficiency of the HD/R Strategy If Bt Plants Produce a Low Toxin Concentration (for One or Two Toxins)

The efficiency of the high toxin concentration for one-toxin *Bt *plants on pest management was experimentally demonstrated in Arizona with a large-scale, long-term (ten years) study. The extensive use of *Bt* cotton had caused regional suppression of *Pectinophora gossypiella*, an ecological cotton specialist [77]. Thus, regions with substantial *Bt* fields imposing high mortality could have negative effects on population growth of a specific pest with a narrow host range before resistance occurs because their diet would be more affected [9,18]. According to Carrière *et al*. [61], the high dose limits the spectrum of resistance mutation conferring resistance in a particular species. Only few mutations could generate sufficient effects to overcome the toxin. The most widely observed mechanism in the laboratory and in the field is related to a reduced binding affinity of Cry toxins to midgut membrane receptors [78].

However, experimental studies have suggested that high Cry toxin concentration significantly increases the development duration of several insect pests [13,46,79,80,81,82,83]. The slowing down of insect development induces an asynchrony in emergence between the refuge and the *Bt *zone [29,84,85,86], which disrupts random mating and leads to assortative mating between adults in each of the two zones [81,86,87]. The reduction of random mating would disturb the effectiveness of the HD/R strategy and some researchers therefore recommend using *Bt *plants with low toxin concentration.

**Figure 4:**
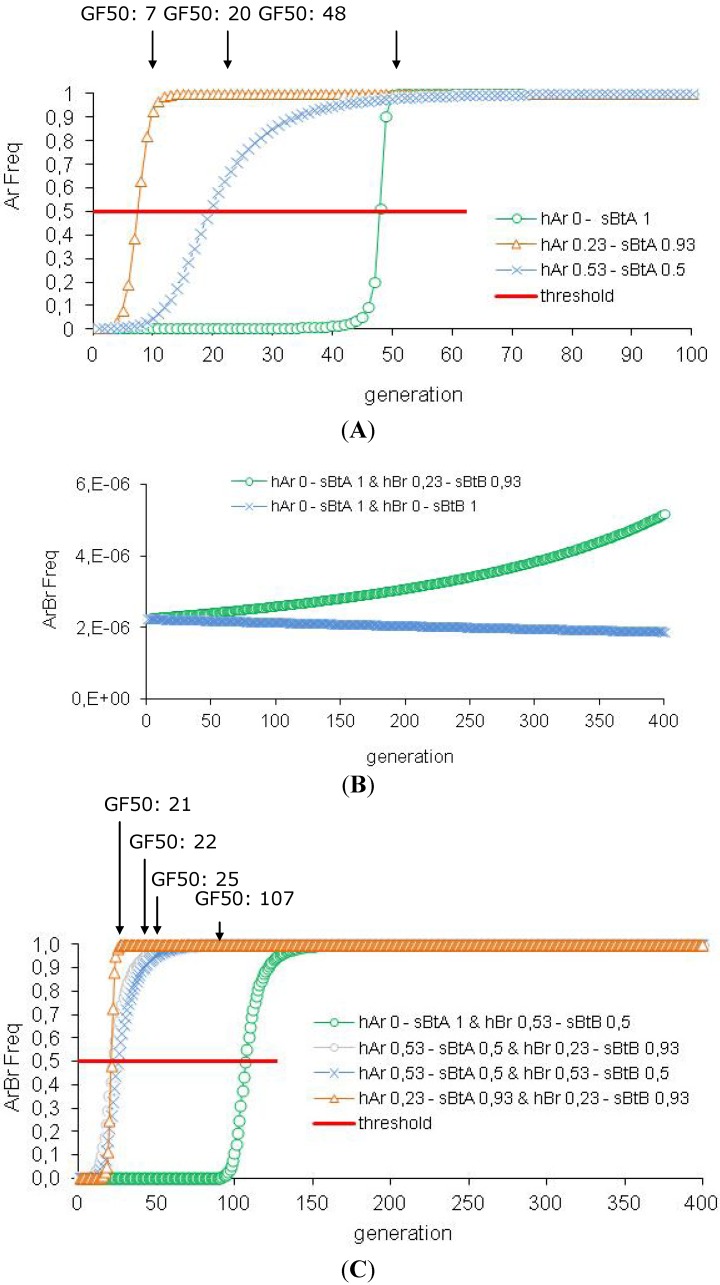
Efficiency of the HD/R strategy with *Bt* plants expressing one or two toxins with low concentration. These simulations are performed for *Bt *plants expressing one or two toxins with initially rare resistance alleles (Ar Freq = Br Freq = 0.0015) and a 5% refuge zone. But the toxin concentration varies (*sBtA* and *sBtB* = 0.50 or 0.93 or 1) involving a variation of the dominance of the resistance allele (*hAr* and *hBr* = 0.53 or 0.23 or 0).

We test the efficiency of the refuge strategy associated with *Bt *plants producing a lower toxin concentration (s*Bt*A: 0.93 or 0.50) with a resistance allele that is not completely recessive (hAr: 0.23 or 0.53, respectively) (see Table 2A and Figure 4A). Our simulations suggest that resistance is no more efficiently controlled for *Bt* plants expressing one toxin than for those expressing two (GF50: 7 and 20, respectively). The pest density is not as reduced after two generations (population decrease: 86% and 39%, respectively) but this difference of population control disappear after ten generations (population decrease: 99.9% and 98.7%, respectively).

Concerning the two-toxin *Bt *plants, we first test the association: one high toxin concentration/one low toxin concentration (see Figure 4B,C and Table 2B). For s*Bt*A: 1-s*Bt*B: 0.93, the GF50 threshold is never reached and for s*Bt*A:1-s*Bt*B: 0.50, GF50 is reached after 107 generations. A 94% population decrease is recorded for these two situations after two generations. Next, we consider *Bt* plants producing the two toxins at lower concentrations and we conclude that less than 25 generations are required to reach GF50 (s*Bt*A = s*Bt*B: 0.53, GF50: 25; s*Bt*A: 0.53-s*Bt*B: 0.93, GF50: 22; s*Bt*A = s*Bt*B: 0.93, GF50: 21). After two generations, the reduction in population density is also not so effective (s*Bt*A = s*Bt*B: 0.53: 67% population decrease; s*Bt*A: 0.53–s*Bt*B: 0.93: 90% population decrease) except for s*Bt*A = s*Bt*B: 0.93 (94% population decrease). However, after ten generations, the population control is efficient whatever the initial condition (100% population decrease). Thus, resistance is efficiently managed when *Bt *plants express at least one toxin at high concentration. We do not recommend producing *Bt *plants with two toxins at low concentrations unless there are no effective alternative methods (biological control, host plant resistance, *etc.*).

### 3.4. Efficiency of the HD/R Strategy If Resistance Is not Rare in the Population and Two-Toxin *Bt* Plants Produce a Low Toxin Concentration

As previously suggested, HD/R strategy applied for two-toxin *Bt *plants is efficient even if the second toxin is produced at low concentration (s*Bt*A: 1 and s*Bt*B: 0.93 or 0.50) or if one resistance allele is rare in the population (0.015 or 0.15). However, what happens if two-toxin *Bt *plants produce one toxin at low titer when a resistance allele is not rare in the population?

**Figure 5:**
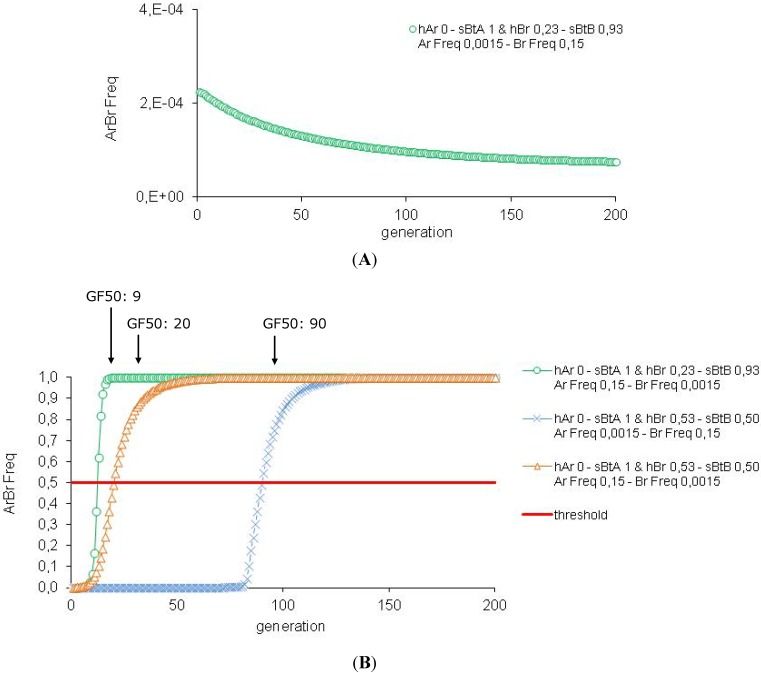
Efficiency of the HD/R strategy when the resistance is not rare in the population and the two-toxin *Bt *plants express different levels of toxin concentration. These simulations are performed for *Bt *plants expressing one or two toxins with 5% refuge zone. The impact of the toxin concentration (*sBtA* = *sBtB* = 1), the variation of the dominance of the resistance allele (*hAr* = *hBr* = 0) and the initial frequency of the resistance alleles (Ar Freq and Br Freq = 0.0015 or 0.015 or 0.15) are tested.

Resistance is still reversed with s*Bt*A: 1-s*Bt*B: 0.93 associated to Ar frequency 0.0015 and Br frequency 0.15 (see Figure 5A,B and Table 2B). Resistance management strategy is also effective with s*Bt*A: 1–s*Bt*B: 0.53 associated to Ar frequency 0.0015 and Br frequency 0.15 (GF50: 90).

However, if the rare resistance allele (0.0015) is not associated with the high toxin concentration (s*Bt*A: 1), resistance management is less viable (see Figure 5B and Table 2B). With s*Bt*A: 1–s*Bt*B: 0.93 associated with Ar frequency 0.15 and Br frequency 0.0015, the threshold GF50 is reached after only nine generations. With s*Bt*A: 1–s*Bt*B: 0.50 associated with Ar frequency 0.15 and Br frequency 0.0015, the threshold is reached after 20 generations. In conclusion, for *Bt *plants expressing one high toxin concentration combined with one low toxin concentration, it is risky to reuse a toxin already produced by a one-toxin *Bt *plant. The resistance allele frequency associated with this “old” toxin may not be rare. Following our results, the rare resistance allele has to be associated with the high toxin concentration for optimal resistance management. Introduction of two new toxins in the plants is recommended when low concentrations are used to limit resistance risk. Moreover, Zhao and colleagues [30] suggest that regulatory agencies consider deregulating one-toxin *Bt *plants as soon as two-toxin *Bt *plants sharing one toxin are available.

Whatever the situation, the population decrease is around 94% after two generations and 100% after ten generations, in every case (see Table 2B).

### 3.5. Efficiency of the HD/R Strategy in Relation to the Refuge Zone Proportion

**Figure 6:**
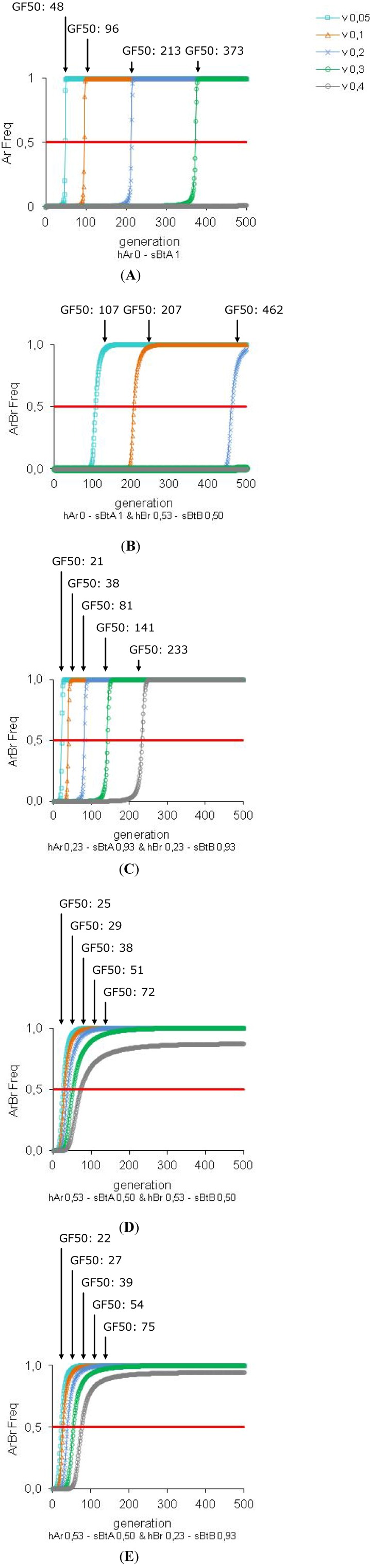
Improvement of the efficie ncy of the HD/R strategy for *Bt *plants with low toxin concentration by increase the refuge zone proportion. These simulations are performed for *Bt *plants expressing one or two toxins with initially rare resistance alleles (Ar Freq = Br Freq = 0.0015). The impact of the variation of the toxin concentration (*sBtA* and *sBtB* = 1 or 0.93 or 0.50), the variation of the dominance of the resistance allele (*hAr* and *hBr* = 0 or 0.23 or 0.53) and the refuge zone proportion (*v *= 0.05 or 0.10 or 0.20 or 0.30 or 0.40) are tested.

A key factor in the HD/R strategy is the refuge zone. The efficiency of the strategy is improved with high refuge zone proportion. The larger is the relative size of the refuge zone, the more insect resistance is delayed (see Figure 6 and Table 2A). However, for *Bt *plants with two toxins at low concentration, resistance is efficiently managed with a 30% refuge zone but the pest population is not so well controlled after two generations (population decrease: 65% for s*Bt*A = s*Bt*B: 0.93; 63% for s*Bt*A: 0.93 and s*Bt*B: 0.53; 45% for s*Bt*A = s*Bt*B: 0.53) (see Figure 6C, Figure 6D and Table 2B). However, after ten generations, we observe an elimination of the pest population whatever the refuge size.

Today, US EPA recommendations allow a small refuge size (5%). The critical question is whether enough susceptible insects will survive in the refuge to provide an effective source of susceptible alleles. As a resistance episode progresses, the refuge zone could be slightly “polluted” by random oviposition. The resistance level of the refuge zone may increase and make it inefficient. However, according to our results, a 5% refuge seems to be sufficient to control resistance. That could increase acceptability of this strategy by growers unwilling to sacrifice a large field area as a refuge. On the other hand, it has been shown that immigration of native susceptible insects could also restore the susceptibility of a population in the *Bt* zone [88]). Counting on immigration of susceptible alleles instead of using refuge zones would be feasible only if immigration patterns of susceptible insects are well known and consistent, and, usually one does not know *a priori *whether such events would occur. Furthermore, extremely high immigration is not compatible with overall pest control objectives (due to the damage caused) and if immigrants are not susceptible, they will not delay resistance [42].

Even though individuals with double resistance may be very rare initially, a refuge is still necessary for two-toxin *Bt* plants to delay resistance (our results, [31,32]). The refuge zone may be costly in terms of yield but the use of two-toxin *Bt *plants with high dose can reduce the amount of refuge required to delay resistance and for extended period in comparison with one-toxin *Bt* plants [31,89]. With a 5% refuge zone, one-toxin *Bt *plants delay resistance during 48 generations (GF50), whereas with two-toxin *Bt* plants, the delay is longer (GF50: never reached in our model). Therefore, two-toxin *Bt* plants have the potential to reduce the requirements for refuges in successful resistance management, which could encourage the industry to develop two-toxin *Bt* plants [90]. However, undersized refuges remain risky, for instance when mortalities of heterozygote insects are lower than expected. The more prudent way to deploy transgenic crops remains to keep refuges as large as economically feasible [31].

## 4. Conclusions

Selection by a regular and frequent use of insecticide inevitably triggers resistance in the target insects. The unique reasonable hope to delay or avoid resistance development is to use practices reducing the intensity of selection pressure. With the HD/R strategy, the refuges reduce toxin selection pressure and the high toxin titer in *Bt* plants purges each generation of as much of the resistance allele as possible. This strategy was developed for *Bt *plants expressing one toxin.

Our model indicates that for *Bt* plants expressing one toxin, a high concentration is an essential condition for resistance management. The pyramided *Bt* plants are more likely to control resistance. One of the two toxins could be expressed at a low concentration, particularly if the two toxins are used for the first time, in which case resistance to these toxins should not be present in the pest population and a small refuge zone would be acceptable.

## References

[1] Crickmore N., Zeigler D.R., Feitelson J., Schnepf E., Van Rie J., Lereclus D., Baum J., Dean D.H. (1998). Revision of the nomenclature for the *Bacillus thuringiensis* pesticidal crystal proteins. Microbiol. Mol. Biol. Rev..

[2] Gill S.S., Cowles E.A., Pietrantonio P.V. (1992). The Mode of Action of *Bacillus thuringiensis* Endotoxins. Annu. Rev. Entomol..

[3] Hofte H., Whiteley H.R. (1989). Insecticidal crystal proteins of *Bacillus thuringiensis*. Microbiol. Mol. Biol. Rev..

[4] Bauer L.S. (1995). Resistance: A threat to the insecticidal crystal proteins of *Bacillus thuringiensis*. Florida Entomol..

[5] Bravo A., Likitvivatanavong S., Gill S.S., Soberón M. (2011). *Bacillus thuringiensis*: A story of a successful bioinsecticide. Insect Biochem. Mol. Biol..

[6] Barton K.A., Whiteley H.R., Yang N.-S. (1987). *Bacillus thuringiensis* §-Endotoxin Expressed in Transgenic *Nicotiana tabacum* Provides Resistance to Lepidopteran Insects. Plant Physiol..

[7] Vaeck M., Reynaerts A., Hofte H., Jansens S., De Beuckeleer M., Dean C., Zabeau M., Montagu M.V., Leemans J. (1987). Transgenic plants protected from insect attack. Nature.

[8] James C. (2010). Global Status of Commercialized Biotech/GM Crops: 2010; ISAAA Brief No. 42.

[9] Gould F. (1998). Sustainability of transgenic insecticidal cultivars: Integrating pest genetics and ecology. Ann. Rev. Entomol..

[10] Caprio M., Summerford D., Simms S. (2000). Evaluating transgenic plants for suitability in pest and resistance management programs. Field Manual of Techniques in Invertebrate Pathology: Application and Evaluation of Pathogens for Control of Insects and Other Invertebrate Pests.

[11] Gould F., Anderson A., Reynolds A., Bumgarner L., Moar W. (1995). Selection and genetic analysis of a *Heliothis virescens* (Lepidoptera: Noctuidae) strain with high levels of resistance to *Bacillus thuringiensis* toxins. J. Econ. Entomol..

[12] Tabashnik B.E., Patin A.L., Dennehy T.J., Liu Y.B., Carrière Y., Sims M.A., Antilla L. (2000). Frequency of resistance to *Bacillus thuringiensis* in field populations of pink bollworm. Proc. Nat. Acad. Sci. USA.

[13] Liu Y.-B., Tabashnik B.E., Dennehy T.J., Patin A.L., Sims M.A., Meyer S.K., Carrière Y. (2001). Effects of *Bt* Cotton and Cry1Ac Toxin on Survival and Development of Pink Bollworm (Lepidoptera: Gelechiidae). J. Econ. Entomol..

[14] Comins H.N. (1977). The development of insecticide resistance in the presence of migration. J. Theor. Biol..

[15] Comins H.N. (1977). The management of pesticide resistance. J. Theor. Biol..

[16] Tabashnik B.E. (1986). Computer simulation as a tool for pesticide resistance management. Pesticide Resistance: Strategies and Tactics for Management.

[17] Tabashnik B.E. (1994). Delaying insect adaptation to transgenic plants: Seed mixtures and refugia reconsidered. Proc. Biol. Sci..

[18] Alstad D.N., Andow D.A. (1995). Managing the Evolution of Insect Resistance to Transgenic Plants. Science.

[19] Storer N.P. (2003). A Spatially Explicit Model Simulating Western Corn Rootworm (Coleoptera: Chrysomelidae) Adaptation to Insect-Resistant Maize. J. Econ. Entomol..

[20] Tabashnik B.E., Van Rensburg J.B.J., Carrière Y. (2009). Field-Evolved Insect Resistance to *Bt* Crops: Definition, Theory, and Data. J. Econ. Entomol..

[21] US EPA Current & Previously Registered Section 3 PIP Registrations. http://www.epa.gov/oppbppd1/biopesticides/pips/pip_list.htm.

[22] Stewart S.D., Adamczyk J.J., Knighten K.S., Davis F.M. (2001). Impact of Bt cottons expressing one or two insecticidal proteins of *Bacillus thuringiensis* Berliner on growth and survival of 8802616noctuid (Lepidoptera) larvae. J. Econ. Entomol..

[23] Mani G.S. (1985). Evolution of resistance in the presence of two insecticides. Genetics.

[24] Tabashnik B.E. (1989). Managing resistance with multiple pesticide tactics: Theory, evidence, and recommendations. J. Econ. Entomol..

[25] Roush R.T. (1994). Managing pests and their resistance to *Bacillus thuringiensis*: Can transgenic crops be better than sprays?. Biocontrol. Sci. Technol..

[26] Livingston M.J., Carlson G.A., Fackler P.L. (2004). Managing Resistance Evolution in Two Pests to Two Toxins with Refugia. Am. J. Agric. Econ..

[27] Gould F., Cohen M.B., Bentur J.S., Kennedy G.G., Van Duyn J. (2006). Impact of small fitness costs on pest adaptation to crop varieties with multiple toxins: a heuristic model. J. Econ. Entomol..

[28] Georghiou G.P., Wirth M.C. (1997). Influence of exposure to single versus multiple toxins of *Bacillus thuringiensis* subsp. israelensis on development of resistance in the mosquito Culex quinquefasciatus (Diptera: Culicidae). Appl. Environ. Microbiol..

[29] Caprio M.A. (1998). Evaluating Resistance Management Strategies for Multiple Toxins in the Presence of External Refuges. J. Econ. Entomol..

[30] Zhao J.-Z., Cao J., Collins H.L., Bates S.L., Roush R.T., Earle E.D., Shelton A.M. (2005). Concurrent use of transgenic plants expressing a single and two *Bacillus thuringiensis* genes speeds insect adaptation to pyramided plants. Proc. Nat. Acad. Sci. USA.

[31] Roush R.T. (1998). Two-toxin strategies for management of insecticidal transgenic crops: Can pyramiding succeed where pesticide mixtures have not?. Philos. Trans. R. Soc. Lond. B Biol. Sci..

[32] Curtis C.F. (1985). Theoretical models of the use of insecticide mixtures for the management of resistance. Bull. Entomol. Res..

[33] Heckel D.G., Gahan L.J., Baxter S.W., Zhao J.Z., Shelton A.M., Gould F., Tabashnik B.E. (2007). The diversity of *Bt* resistance genes in species of Lepidoptera. J. Invertebr. Pathol..

[34] Monsanto website. http://monsanto.mediaroom.com/.

[35] Downes S., Parker T., Mahon R. (2010). Incipient Resistance of *Helicoverpa punctigera* to the Cry2Ab *Bt* Toxin in Bollgard II^®^ Cotton. PLoS One.

[36] Georghiou G.P., Taylor C.E. (1977). Genetic and biological influences in the evolution of insecticide resistance. J. Econ. Entomol..

[37] Introduction to Biotechnology Regulation for Pesticides< |Pesticides| US EPA. http://www.epa.gov/oppbppd1/biopesticides/regtools/biotech-reg-prod.htm#crops.

[38] Guse C.A., Onstad D.W., Buschman L.L., Porter P., Higgins R.A., Sloderbeck P.E., Cronholm G.B., Peairs F.B. (2002). Modeling the development of resistance by stalk-boring Lepidoptera (Crambidae) in areas with irrigated transgenic corn. Environ. Entomol..

[39] Andow D.A., Olson D.M., Hellmich R.L., Alstad D.N., Hutchison W.D. (2000). Frequency of resistance to *Bacillus thuringiensis* toxin Cry1Ab in an Iowa population of European corn borer (Lepidoptera: Crambidae). J. Econ. Entomol..

[40] Liu Y.B., Tabashnik B.E., Meyer S.K., Carrière Y., Bartlett A.C. (2001). Genetics of pink bollworm resistance to *Bacillus thuringiensis* toxin Cry1Ac. J. Econ. Entomol..

[41] Vacher C., Bourguet D., Rousset F., Chevillon C., Hochberg M.E. (2003). Modelling the spatial configuration of refuges for a sustainable control of pests: A case study of *Bt* cotton. J. Evol. Biol..

[42] Tabashnik B., Gould F., Carriere Y. (2004). Delaying evolution of insect resistance to transgenic crops by decreasing dominance and heritability. J. Evolution. Biol..

[43] Carriére Y., Ellers-Kirk C., Liu Y.B., Sims M.A., Patin A.L., Dennehy T.J., Tabashnik B.E. (2001). Fitness costs and maternal effects associated with resistance to transgenic cotton in the pink bollworm (Lepidoptera: Gelechiidae). J. Econ. Entomol..

[44] Carrière Y., Ellers-kirk C., Patin A.L., Sims M.A., Meyer S., Liu Y., Dennehy T.J., Tabashnik B.E. (2001). Overwintering cost associated with resistance to transgenic cotton in the pink bollworm (Lepidoptera: Gelechiidae). J. Econ. Entomol..

[45] Sayyed A.H., Wright D.J. (2001). Fitness costs and stability of resistance to *Bacillus thuringiensis* in a field population of the diamondback moth *Plutella xylostella* L. Ecol. Entomol..

[46] Bird L.J., Akhurst R.J. (2004). Relative fitness of Cry1A-resistant and -susceptible *Helicoverpa armigera* (Lepidoptera: Noctuidae) on conventional and transgenic cotton. J. Econ. Entomol..

[47] Conner J.K., Hartl D.L. (2004). A Primer of Ecological Genetics.

[48] Gould F., Martinez-Ramirez A., Anderson A., Ferre J., Silva F.J., Moar W.J. (1992). Broad-spectrum resistance to *Bacillus thuringiensis* toxins in *Heliothis virescens*. Proc. Nat. Acad. Sci..

[49] McGaughey W.H., Johnson D.E. (1994). Influence of Crystal Protein-Composition of *Bacillus-Thuringiensis* Strains on Cross-Resistance in Indianmeal Moths (Lepidoptera, Pyralidae). J. Econ. Entomol..

[50] US EPA *Bt* Plant-Pesticides Biopesticides Registration Action Document. http://www.epa.gov/scipoly/sap/meetings/2000/october/brad4_irm.pdf.

[51] Onstad D.W., Meinke L.J. (2010). Modeling evolution of *Diabrotica virgifera* virgifera (Coleoptera: Chrysomelidae) to transgenic corn with two insecticidal traits. J. Econ. Entomol..

[52] Shelton A.M., Zhao J.Z., Roush R.T. (2002). Economic, ecological, food safety, and social consequences of the deployment of *Bt *transgenic plants. Ann. Rev. Entomol..

[53] Monsanto proposes natural refuge for Bollgard II cotton, Management content from Delta Farm Press. http://deltafarmpress.com/management/monsanto-proposes-natural-refuge-bollgard-ii-cotton.

[54] US EPA EPA Approves Natural Refuge for Insect Resistance Management in Bollgard II Cotton. http://www.epa.gov/oppfead1/cb/csb_page/updates/2007/bollgard-cotton.htm.

[55] Tabashnik B.E., Liu Y., Malvar T., Heckel D.G., Masson L., Ferre J. (1998). Insect resistance to *Bacillus thuringiensis*: Uniform or Diverse?. Phil. Tran. R. Soc. Lond. Series B Biol. Sci..

[56] Frutos R., Rang C., Royer M. (1999). Managing insect resistance to plants producing *Bacillus thuringiensis* toxins. Crit. Rev. Biotechnol..

[57] Rawlings P., Davidson G., Sakai R.K., Rathor H.R., Aslamkhan M., Curtis C.F. (1981). Field measurement of the effective dominance of an insecticide resistance in anopheline mosquitos. Bull. World Health Organ..

[58] Sayyed A.H., Haward R., Herrero S., Ferre J., Wright D.J. (2000). Genetic and Biochemical Approach for Characterization of Resistance to *Bacillus thuringiensis* Toxin Cry1Ac in a Field Population of the Diamondback Moth, *Plutella xylostella*. Appl. Environ. Microbiol..

[59] Bourguet D., Prout M., Raymond M. (1996). Dominance of insecticide resistance presents a plastic response. Genetics.

[60] Raymond B., Sayyed A.H., Wright D.J. (2005). Genes and Environment Interact to Determine the Fitness Costs of Resistance to *Bacillus thuringiensis*. Proc. R. Soc. B Biol. Sci..

[61] Carriére Y., Tabashnik B. (2001). Reversing insect adaptation to transgenic insecticidal plants. Proc. R. Soc. Lond. Series B Biol. Sci..

[62] Vacher C., Bourguet D., Rousset F., Chevillon C., Hochberg M.E. (2004). High dose refuge strategies and genetically modified crops–reply to Tabashnik *et al.*. J. Evol. Biol..

[63] Lenormand T., Raymond M. (1998). Resistance management: The stable zone strategy. Proc. Biol. Sci..

[64] Orr D.B., Landis D.A. (1997). Oviposition of European corn borer (Lepidoptera: Pyralidae) and impact of natural enemy populations in transgenic *versus* isogenic corn. J. Econ. Entomol..

[65] Ramachandran S., Buntin G.D., All J.N., Tabashnik B.E., Raymer P.L., Adang M.J., Pulliam D.A., Stewart C.N. (1998). Survival, development, and oviposition of resistant diamondback moth (Lepidoptera: Plutellidae) on transgenic canola producing a *Bacillus thuringiensis* toxin. J. Econ. Entomol..

[66] Anonymous The R Project for Statistical Computing. http://www.r-project.org/.

[67] Tabashnik B.E., Croft B.A. (1982). Managing pesticide resistance in crop-arthropod complexes: Interactions between biological and operational factors. Environ. Entomol..

[68] Mallet J., Porter P. (1992). Preventing insect adaptation to insect-resistant crops: Are seed mixtures or refugia the best strategy?. Proc. Biol. Sci..

[69] Crow J.F., Kimura M. (1970). An Introduction to Population Genetic Theory.

[70] Ricker W.E. (1954). Stock and recruitment. J. Fish. Board Can..

[71] Gould F. (2003). *Bt*-resistance management-theory meets data. Nat. Biotechnol..

[72] Tabashnik B.E., Gassmann A.J., Crowder D.W., Carrière Y. (2008). Insect resistance to *Bt* crops: Evidence *versus* theory. Nat. Biotechnol..

[73] Luttrell R.G., Ali I., Allen K.C., Young S.Y., Szalanski A., Williams K., Lorenz G., Parker C.D., Blanco C. (2004). Resistance to Bt in Arkansas populations of cotton bollworm. Proceedings of 2004 Beltwide Cotton Conferences.

[74] Ali M.I., Luttrell R.G., Young S.Y. (2006). Susceptibilities of *Helicoverpa zea* and *Heliothis virescens* (Lepidoptera: Noctuidae) populations to Cry1Ac insecticidal protein. J. Econ. Entomol..

[75] Matten S.R., Head G.P., Quemada H.D., Romeis J., Shelton A.M., Kennedy G.G. (2008). How governmental regulation can help or hinder the integration of *Bt* crops within IPM programs. Integration of Insect-Resistant Genetically Modified Crops withinIPM Programs.

[76] Van Rensburg J.B.J. (2007). First report of field resistance by stem borer, *Busseola fusca* (Fuller) to *Bt*-transgenic maize. S. Afr. J. Plant Soil.

[77] Carrière Y., Ellers-Kirk C., Sisterson M., Antilla L., Whitlow M., Dennehy T.J., Tabashnik B.E. (2003). Long-term regional suppression of pink bollworm by *Bacillus thuringiensis* cotton. Proc. Nat. Acad. Sci. USA.

[78] Van Rie J., McGaughey W.H., Johnson D.E., Barnett B.D., Van Mellaert H. (1990). Mechanism of insect resistance to the microbial insecticide *Bacillus thuringiensis*. Science.

[79] Giles K.L., Hellmich R.L., Iverson C.T., Lewis L.C. (2000). Effects of transgenic *Bacillus thuringiensis* maize grain on *B. thuringiensis*-susceptible *Plodia interpunctella* (Lepidoptera: Pyralidae). J. Econ. Entomol..

[80] Sedlacek J.D., Komaravalli S.R., Hanley A.M., Price B.D., Davis P.M. (2001). Life History Attributes of Indian Meal Moth (Lepidoptera: Pyralidae) and Angoumois Grain Moth (Lepidoptera: Gelechiidae) Reared on Transgenic Corn Kernels. J. Econ. Entomol..

[81] Liu Y.-B., Tabashnik B.E., Dennehy T.J., Patin A.L., Bartlett A.C. (1999). Development time and resistance to *Bt* crops. Nature.

[82] Lopez M.D., Sumerford D.V., Lewis L.C. (2010). Nosema pyrausta and Cry1Ab-incorporated diet led to decreased survival and developmental delays in European corn borer. Entomologia Experimentalis et Applicata.

[83] Gryspeirt A., Grégoire J.-C. (2012). Effects of Two Varieties of *Bacillus thuringiensis* Maize on the Biology of *Plodia Interpunctella*. Toxins.

[84] Storer N.P., Van Duyn J.W., Kennedy G.G. (2001). Life history traits of *Helicoverpa zea* (Lepidoptera: Noctuidae) on non-*Bt* and *Bt* transgenic corn hybrids in eastern North Carolina. J. Econ. Entomol..

[85] Horner T.A., Dively G.P., Herbert D.A. (2003). Effects of MON810 *Bt* field corn on adult emergence of *Helicoverpa zea* (Lepidoptera: Noctuidae). J. Econ. Entomol..

[86] Peck S.L., Gould F., Ellner S.P. (1999). Spread of resistance in spatially extended regions of transgenic cotton: implications for management of *Heliothis virescens* (Lepidoptera: Noctuidae). J. Econ. Entomol..

[87] Fabrick J.A., Forlow Jech L., Henneberry T.J. (2009). Novel pink bollworm resistance to the *Bt* toxin Cry 1Ac: Effects on mating, oviposition, larval development and survival. J. Insect Sci..

[88] Shelton A.M., Tang J.D., Roush R.T., Metz T.D., Earle E.D. (2000). Field tests on managing resistance to *Bt*-engineered plants. Nat. Biotech..

[89] Gould F. (1991). The evolutionary potential of crop pests. Am. Sci..

[90] Zhao J.Z., Cao J., Li Y., Collins H.L., Roush R.T., Earle E.D., Shelton A.M. (2003). Transgenic plants expressing two *Bacillus thuringiensis* toxins delay insect resistance evolution. Nature Biotechnol..

